# Participative epidemiology and prevention pathway of health risks associated with artisanal mines in Luhihi area, DR Congo

**DOI:** 10.1186/s12889-023-15020-3

**Published:** 2023-01-18

**Authors:** Christian Ahadi Irenge, Parfait Kaningu Bushenyula, Emmannuel Bayubasire Irenge, Yves Coppieters

**Affiliations:** 1 Université Officielle de Bukavu, Bukavu, Democratic Republic of the Congo; 2 Angaza Institute, Institut Supérieur de Développement Rural, Bukavu, Democratic Republic of the Congo; 3 School of Public Health, Université Libre de Bruxelles (ULB), Brussels, Belgium

**Keywords:** Artisanal mining, Health risks, Participative epidemiology, And prevention dialog for change

## Abstract

**Background:**

Health issues are associated with artisanal mining in the DR Congo. The scenario is worst when artisanal mining is done informally or with limited material and technical resources.

This paper argues that the adoption of healthy practices by artisanal miners might be limited given that it involves unrealistic socio-economic, and administrative aspects and access to health risk prevention means.

Making a conceptual framework on the feasibility of revolutionizing artisanal mining practices linked to health risks in the DR Congo requires trans-disciplinary interventions and researches. This case study aims at co-analyzing with actors in the Luhihi artisanal gold mine, the epidemiology of health issues. It also aims at describing the dynamics of resources that mining actors mobilize or think they can mobilize in order to prevent health risks.

**Methods:**

A “socio-anthropological” qualitative study with “transdisciplinary methods” was carried out the Luhihi artisanal mining. Data collection tools and methods included an exploratory survey, semi-structured interviews. Focus groups (FG) mixed with proportional piling were used to support the open-ended interview discussions. The actors interviewed were selected by “convenience sampling” and the saturation principle indicated the size of the sampling. In total, 67 persons were interviewed and 5 FG each consisting of 5 to 10 mining actors were organized. Data were triangulated among respondents to ensure their veracity and an “inductive thematic data analysis” was applied.

**Results:**

Key findings are the role of actors involved the organization system at the Luhihi artisanal mining site; a description of a participative epidemiology and determinants of health issues; presentation of the importance of health risks as perceived by mining actors; the constraints in the common illenesses treatment; and opportunities of collective actions for gathering resources required for the organization of healthcare services.

**Conclusion:**

The results are translated into a grid of powers and interests in relation to the mobilization of resources for the prevention and treatment of health issues. The dialogue for change regarding the ignorance of the actors to exposure to chemical risks such as to exposure mercury, silica, carbon monoxide, and cyanide also entailed the translation of the results. In addition, an analysis of the ability of artisanal mining actors to implement health risk prevention services was made.

## Introduction

Artisanal mining is a crucial source of income for rural communities especially in developing countries. Besides agriculture, artisanal mining is the most important activity for rural economy in sub-Saharan Africa [1]. Researches from various disciplines have demonstrated that urban and rural Africans take up mining as a response to unemployment, lack of credit and poor income prospects in the agricultural sector. In addition, mining provides young people with the ability to achieve a certain degree of personal autonomy [2]. Many studies have captured the synergies between artisanal small-scale mining (ASM) and farming in sub-Saharan Africa. An examination of dynamic strategies of land recipients in Zimbabwe shows that farmers, in mineral rich regions, consciously engage in ASM as part of their livelihood provisioning and some farmers perceived ASM as a way to secure tenure on their landholdings [3]. In Mozambique, proceeds from small-scale gold mining at Chazuka have enabled individuals to buy fertilisers and other crucial farm inputs. In Liberia, many farm families grow rice to attract and feed labourers recruited specifically to mine for diamonds. In Cameroon, where poor markets and impenetrable forest constrain agricultural development, ASM is largely seasonal but is increasingly important for incomes [4].

Although practices in ASM vary around the world, it is recognized that artisanal mining frequently creates environmental, social and health problems. This is due to the informal nature of the artisanal mining sector, where national standards for health and safety are often not applicable [5, 6]. Formalisation of the sector of ASM is expected to bring miners access to technical support, credit, and clear property and user rights. This might help governments minimise the negative social and environmental impacts of ASM while deriving revenue from their mineral resources [7]. In sub-Saharan Africa, there are a range of initiatives that have been setted to regulate and formalise the artisanal mining sector. However, evidence showed that they have failed to have a positive impact [8]. A case study has examined the implementation of the Ghanaian ASM formalisation from 2017 to 2020. It found that despite the repetitive enactment of the ASM formalisation discourse by the state, informality within ASM persists. That’s because of the visible display of practical norms enacted by myriad political actors with competing political and material interests [9]. Findings have revealed that despite showing considerable promise at first, the drive to formalize ASM in Mozambique has lost considerable momentum. A bureaucratic licensing scheme, overlapping responsibilities at the ministry of mineral resources and energy, and a shortage of information about miners have contributed to this slowdown [4]. Focusing on Liberia, Maconachie and Conteh have argued that the persistence of informality in the sector needs to first be understood as a rational strategy for those who profit from it. Only then can sustainable mining reforms be linked to broader national and international extractive sector policy frameworks [10]. The concept “extralegality” of the ASM formalization is applied to the case of Uganda. It is argued that to make formalization work, miners must also be “capitalized” in ways that permit them to move from transient artisanal mining, to more sustainable small- and medium-scale mining [11]. Some factors hindering the effective implementation of the initiatives of ASM formalisation in DR Congo include among others; the state capacity and political will; the complex dynamics of power and relations among actors in the current system of artisanal mining and trade [12].

The DR Congo harbors approximately two million ASM workers and occupies the third place worldwide in terms of the absolute number of people working in ASM. The eastern part of the country hosts at least 382,000 artisanal miners across 2700 sites, and the subsistence of millions of people depends on artisanal mining [13, 14]. Despite an emerging industrial production, much of the mineral production and trade is still artisanal and not formalized, especially for gold. Moreover, during the war (1998–2003), the region’s minerals have attracted the greed of rebel groups, foreign occupying forces as well as the national army, which has resulted in massive human right abuses and violence. Yet despite its informality, artisanal mining contributes to millions of individual livelihoods as well as to broader development in eastern the country [8].

In the DR Congo, many work-related and health risks are associated with ASM, especially when it is practiced informally or with limited material and technical resources [15]. Health risk issue needs to be thoroughly studied. It contributes to the incompleteness of the current DR Congo mining code which does not clearly and comprehensively provide the safety and health risk prevention standards for artisanal miners. Indeed, the reform of the Mining Code of the DR Congo adopted in 2002 introduces the obligation to respect the standards in terms of hygiene and safety in the Artisanal Mining Zones. To achieve this, the Mining Code refers the implementation of these standards to a specific regulation which is yet to exist [16].

Health risks and hazards from artisanal mining are categorized as chemical, biological, biomechanical, physical and psychosocial. (i) Chemical risks include exposure to mercury, silica, arsenic, lead, methane, sulfur dioxide, nitrous oxide, carbon monoxide and cyanide among others [17]. Evidences from a case study of Kamituga Artisanal Mining in the DR Congo show case the practice of gold amalgamation by using mercury [18]. Elemental mercury intoxication is associated with neurological, kidney and autoimmune impairment. High inhalational exposures can lead to respiratory failure and death. Organic mercury intoxication hinders motor and mental development [19]. The Congolese mining regulations prohibit the use of mercury outside licensed plants. However, those regulations are not enforced because of fact that public and technical services that are mandated to protect the environment are subject to several financial, material, technical and human constraints [18].

(ii) Biological risks include exposure to sexually transmitted infections as well as exposure to pathogenic microorganisms such as cholera, malaria, dengue causative agents among others [20]. A study on “Social impacts of artisanal cobalt mining in Katanga, DR Congo” reported that in camps, there is very little effort made to insure sanitation. No latrines are available, quality of drinking water is poor, and human waste management is usually ad hoc. As a consequence, the healthiness of mining communities is deteriorating with higher prevalence rate of HIV, diarrhoea, hepatitis, meningitis, bilharziosis, cholera, typhoid fever, tetanus, typhus, malaria, yellow fever, tuberculosis, etc. [21].

(iii) Biomechanical risks include overworking and exposure to factors causing musculoskeletal disorders, trauma, etc. [22] (iv) In addition, there are physical risks such as heat, humidity, oxygen levels, etc. (v) Psychosocial risks include alcohol and drug abuse, stress and fatigue [23, 24]. For instance, in artisanal cobalt mining in the eastern DR Congo tunnels are longer than 30 m. Hundreds or even thousands of miners often dig in tiny pits, with neither coordination nor knowledge of previous operations that may affect ground stability. Therefore, the danger of landslide is permanent. The exact number of accidents has not yet been exhaustively reported given that reporting a potential danger of landslide from a given mine may lead to its closure by authorities [21].

In eastern DR Congo, several research projects have been conducted to characterize and assess the risks of using chemical agents in artisanal mining on health, ecology and agriculture. These include a study on the effects of heavy metals on health and the environment; a study on environmental risks and respiratory health; a study on the mining sector and heavy metal pollution; a study on environmental change related to artisanal mining, etc. Besides, in partnership with government agencies, research findings on chemical agents in artisanal mining have been translated into a National Action Policy in the DR Congo [14, 18, 23, 25].

**Fig. 1:**
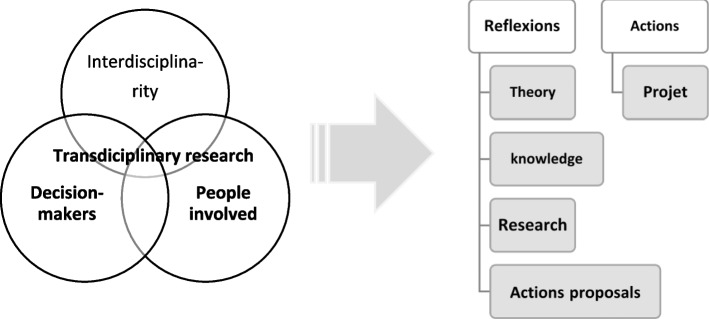
Scheme of the transdisciplinary approaches

The adoption of health risks prevention practices in the artisanal mining sector faces several socio-economic and administrative issues that may limit their adherence and access by artisanal miners. Therefore, in order to optimize the translation of existing research insights into societal change, it is necessary to mobilize “trans-disciplinary investigation on the subject”. “ Transdisciplinary research (Fig. 1) brings together scientists, policy makers, and concerned people to analyze problems, discuss about what they consider to be a desirable future, and develop concrete strategies and actions that may support needed changes” [26, 27]. This investigation aims at co-producing with the stakeholders the impact pathway of preventives actions related to health risks in artisanal mining in the DR Congo. It has been done by applying participatory methods and translating research findings into perceptive actions. These integrate socio-economic contexts, interests and power of mining actors in addition to the key role of mining regulation agencies.

Without addressing the various socio-anthropological components of mine actors, this report presents observations from a study conducted in November 2021 on participatory epidemiology. Besides, it addresses the dynamics of preventive health resources at the Luhihi artisanal gold mining, in the South Kivu, DR Congo.

## Methodology

### Study aim

This socio-anthropological study was conducted with the aim of generating together with the actors of the Luhihi gold mine, their perceptions and the importance they give to health risks. This study also aimed at describing the dynamics of the resources of health risk prevention which the actors mobilize or may be able to mobilize.

### Study setting

The Luhihi mining site is located at the locality of Luhihi, in the Kabare territory, in South Kivu Province, in the DR Congo, at about 50 km north of Bukavu town. Soon after the discovery of Luhihi mining site by the local population in 2019, many diggers settled in Luhihi. The cholera outbreak that occurred at the mine site in September 2020 illustrates one of the environmental health issues.

The division of mining of the South Kivu Province declared that the miners through the COMILU cooperative illegally carry out their artisanal gold mining activities in a mining square which has no national approval, In addition, they operate in a site that is not qualified in accordance with article 114 of the mining code. The current mining code in the DR Congo, recommend mining cooperatives to operate in Artisanal Exploitation Zone (AEZ). AEZs are areas exclusively designated for artisanal mining activities. The formation of an AEZ is restricted to areas where ‘the technological and economic factors are not suited for industrial exploitation’ [28]. To establish control over the operation, in May 2021, a decree from the government of the South Kivu province suspended all mining activities. Miners, traders and members of armed forces were required to leave the sites and mining activities resumed 2 months later.

### Study design and participatory approaches

**Fig. 2:**
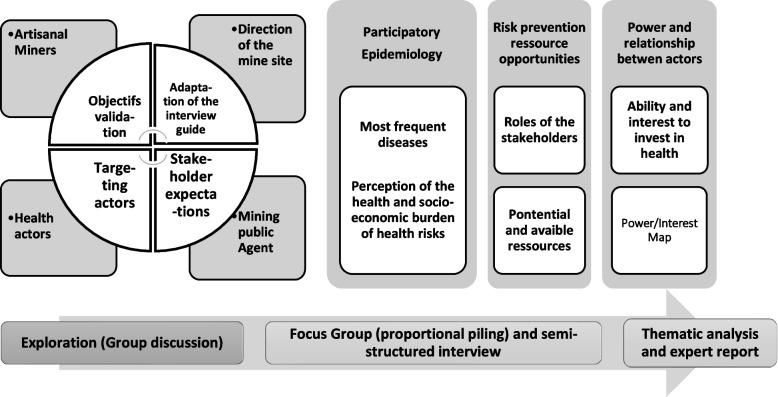
Scheme of the methodological approach of the investigation

A qualitative study through a “ socio-anthropological “ survey [29] was conducted and consisted of an immersion in the study environment and a participative interaction with the concerned social actors to elucidate the research questions (Fig. 2). Data collection tools and methods used included an exploratory survey, semi-structured interviews, and focus groups mixing a proportional piling to support the open-ended interview discussions. In addition, we mobilized a “transdisciplinary approach” [27] aiming at integrating the local knowledge of the actors in the study area. With this integration objective insight, an exploratory survey with key actors allowed us to co-produce the objectives of the investigation and to adapt the interview guide. The discussion themes of the focus groups were produced from the observations of the semi-structured interviews (Fig. 2). We did an “ inductive thematic data analysis “ [30, 31]. The survey results were transcribed and presented by identifying recurring themes from the data (Fig. 2).

### Participant sampling

The interviewed actors were selected by “convenience sampling” [32]. Firstly, the actors were selected based on their availability and their willingness to participate in the study. Secondly, the informants were targeted based on the information that we aimed to collect from them in relation to their expertise and their personal experiences. The “saturation principle” indicated the size of the sampling by assessing the redundancy of the data. To ensure the veracity of the information and to manage the production of concerted information, we “triangulated the opinions” collected by the various actors [33].

### Exploratory interview guides

During the exploratory investigation we used “the consultation approaches” [34] to make key actors participate in the study design and to integrate their understanding in the problem framing. The participants were the Mining camp management agents, “PDG”, Miners, and Economic actors. These investigations aimed at (i) validating the study objectives, (ii) selecting and understanding the expectations of the actors, (iii) and testing and adapting the investigation tools. The exploratory investigation was based on convenience sampling and we used the participatory meeting and brainstorming tools (Table 1). “This tool consisted in gathering a group of actors so that they collectively produce a maximum of new ideas on a given theme. This is followed by the selection of pertinent information provided [34].

**Table 1:** Exploratory interviews with key stakeholders

Investigation tools	Subjects	Participants
Brainstorming Participatory meeting	Description of the study setting, Targeting of the actors implicated in health care services, Validation of the research objectives in link with the field context, Testing and adapting of the interview guide.	Mining camp management agents “PDG” Miners Economic actors

### Semi-structured interviews

A total of 67 people was interviewed at the Luhihi gold mining using an open-ended interview guide lasting a maximum of 1 h (Table 2).

**Table 2:** Participants in semi-structured interviews

Profils	Number	Women	Men
Camp commity agents	2		2
State Mining Agent	6		6
« PDG »	5		5
Miners	25		25
Economic actors	16	7	9
Sex Professionals	13	13	
Total	67	20	47

### Focus groups

**Fig. 3:**
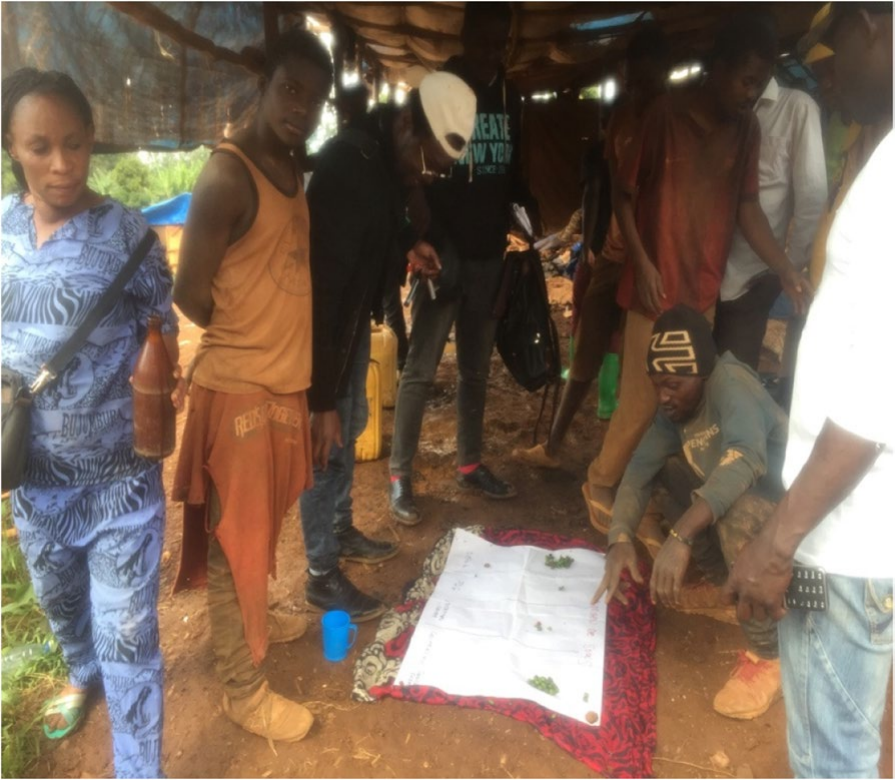
Focus group conducted with the proportional piling tool

We organized 5 Focus Groups (Fig. 3) with 5 to 10 participants lasting a maximum of 1 h and half (Table 3). As an integration aspect, the themes of the FG guide were developed based on the data collected from the semi-structured interviews. The FGs were facilitated using proportional piling with the added value of being a participatory, insertion and semi-quantitative data collection tool.

**Table 3:** Characteristics of the FG

	Participants	Nomber	Women	Men
FG1	Sex Professionnals	5	5	
FG2	Economics actors	4	1	3
	Mining Camp Management Officer	1		1
FG3	Economics actors	8	3	5
FG4	Artisanal diggers	7		7
	Economic Actors	2	2	
	“PDG”	1		1
FG5	Artisanal diggers	6		6
	“PDG”	3		3
Total		37	11	26

### Interview guide

During the semi-structured interview and focus groups guides we used open questions on the subject of participatory epidemiology, availability and access to health care, initiative of collective actions for care as well as the power dynamics and actors’ interests in mobilizing health care resources (Table 4).

**Table 4:** Themes of the Interview guide

General themes	Sub-themes
Participatory epidemiology	- Most frequent pathology - Vernacular names of diseases and their illustration by symptoms - Importance of diseases according to mortality - Importance of the disease according to the difficulty of treatment
Availability and access to health care	- Health care structures - Different health care itineraries - Different health care options - Determinants of the choice of health care options - Sources of income for health care
Initiative of collective actions for care	- Willingness to participate in collective actions like assurances - Potential actors well placed for initiation
Power dynamics and actors’ interests in mobilizing health care resources	- Actors with interest in action - Actors with power of action - Actors needing to be motivated for acting - Alignment of stakeholders’ interests

### Ethical considerations

During field data collection; local traditions, folk customs, norms and values have been respected. In addition, the ethical principles of respect for persons, respect for the interests of justice of respondents, and respect for communities have been observed. Respondents consciously gave their consent before the interviews begun. During the data collection process, we faced ethical considerations related to the conditions and the expectations of the respondents. Some of the respondents requested for some money and sometimes for some beer in order to participate the interviews. We managed these in an amicable way taking into consideration the opinion of the respondents. Furthermore, some of the informants expected us to introduce NGOs as an output of this research. The respondents were ensured that the unique objective of this research is to collect information and to disseminate the results and recommendations.

## Results

### Identification of actors in the system of interest

The Luhihi artisanal mine covers several mountains located in Luhihi Health Subarea, Katana Health Zone and Kabare territory in South Kivu. The state health system organizes a reference Hospital for the Katana Health Zone which gather 18 Health Areas each with a Health Center, including Luhihi. With the discovery of the mining in 2019, around 1000 households of diggers have settled on the Luhihi site. The habitations are of poor quality and the surrounding environment has unfavorable hygiene and sanitary conditions. The conditions spontaneously resulted from the installation of the mine. The habitations are made up of straw and plastic sheeting while the toilets are made up of different types of materials and are not adequately supplied with water. The main source of water is the river that overhangs the hill and is also used for the gold panning. On the mining site there are around 10 small shops from which drugs are acquired on the basis of self-medication. The shops are owned by shopkeepers without health training. In addition, medical treatments and hospitalization are provided in around three buildings each made up of plastic falls without basic equipment (Fig. 6). A shopkeeper, who runs a drugstore, revealed that “dispensaries at the mine site provide medical care for all common illnesses. In case of necessity of blood perfusion, the rescue often comes from the Health Center of the Health Area of Luhihi”.

Health risks related to artisanal mining is a societal challenge involving many actors with different roles, interests, and power. Understanding this is important as specify the people who should be involved in the research and project actions. The actors (persons, groups, or organizations) acting at the Luhihi gold mining site were identified and a clear image of their role related to the mining activities was established (Table 5).

**Table 5:** Actors in the system of interest

Actor designations	Specifications
Camp Committee	Non-state structure that facilitates the management of the various interactions between actors in the mining area. The structure is appointed by the actors in the mining site.
Mining cooperatives	Mining-focused organizations recognized by the state authorities, owned and controlled by their members to meet their common economic, social and cultural needs and aspirations.
Mining agents	Agents from the Ministry in charge of mining who control mining operations
Mine Police	State police services
« PDG »	People authorized by the mining cooperative to exploit minerals from the mining site. They make verbal work agreements with the diggers, avail resources and make the follow up of the exploitation works.
« Supporters »	They provide additional financial resources to the PDG when it is required.
Diggers called « Demushimba »	In charge of manual drilling of underground mining galleries.
Transportors	People in charge of carrying the rocks extracted from the underground pipes to the crushers.
Actors in charge of crushing and « washing »	People in charge of reducing the size of rocks by washing with water and mercury.
Economic actors	Owners of health centers, pharmacies, restaurants, bars, recreational activities and shops selling basic necessities.
Professionals of sex called «Diggers without spade”.	Sex Professionals
Natives	Inhabitants of the mine site and surrounding areas before the mining began

### Contribution to participatory epidemiology of health risks agents ansd effects

The importance of health risks and diseases was assesseded in terms of perception of level of danger that the risk agent bears, the frequency of occurrence of consequences related to the risk, morbidity and mortality of the disease, and accessibility of treatment of the disease (Table 6).

**Table 6:** Perceived importance of health risks and potential resources for preventive and curative medical care

Respondents	Perception of health risks and diseases that are important	Perceived potential resources reported for health risk prevention and curative medical care
Camp Committee	Malaria, Cholera epidemic in 2020 in the mining site.	Individual resources, actions of few NGOs, No health intervention from the office of Health Zone.
Drug sellers	Gonorrhea, HIV/AIDS.	Individual resources, No health interventions by NGOs, No health interventions from the office of Health Zone.
Diggers called « Demushimba »	Tiredness, Washa^a^ Gonorrhoea, Reference to Cholera Epidemic.	Use of medicinal plants in case of scarcity of medicines. Some “PDGs” and “supporters” take in charge the health care of diggers and others do not. Savings or commercial revenues in the family of the digger. Collective contributions by diggers, before finding the mineral, the care can be supported by “PDG and supporters” and after obtaining the mineral, the care is provided by the diggers themselves.
« PDG »	Malaria, Cold, Diarrhea.	Individual resources
Mining agents	Reference to Cholera Epidemic in 2020	Individual resources
Sexe professionnals	Washa, Blennorrhagia Diarrhea Malaria Chamuyangu^b^ Lubenji^c^	Individual resources, Distribution of condoms by an NGO through the Head mother ^d^
Economic actors	Typhoid fever Gonorrhoea Malaria	Individual Resources

^a^Washa: a symptom described as a sexually transmitted infection characterized of rashes all over the body and itching

^b^Chamuyangu: a symptom described with rashes on the stool and coughing

^c^ Lubenji: a symptom characterized with migrating skin rashes on the body and itching

^d^ Head mother: representative of female sex professionals

From the above, the mining actors give more attention to biological and biomechanical health risks than other risk agents (physico-chemical and psychological among others). In addition, epidemiological data from health centers in the Luhihi health area indicate that the diseases with the highest incidences are malaria, acute renal failure, thyphoid fever, bloody diarrhea, cholea, measles [35].

### Determinants of biological health risks

In Luhihi, the major determinants of the recrudescence of biological risks and pathologies (such us malaria, diarrheal diseases, thyphoid fever, etc.) are mainly the precarious hygiene, sanitation conditions and the scarcity of potable drinking water and to adequate health care services such as an antibiotic treatment. The tropical climate and the stagnant water around the junk houses are conducive for malaria development. The Cholera outbreaks that occured in September 2020 at the Luhihi mine site were favored by the lack of toilets, the defecation in the nature, the inaccessibility to potable drinking water, etc. The prevalence of sexually transmitted diseases is maintained by unprotected sex and lack of information. Furthermore, the mining site is always a vestive environment where on one hand, diggers want to enjoy the money they earn after risking their own lives by entering the underground in search for gold. On the other hand the availability of sex partners (called “spadeless dealers”) who are harbored and kept by the owners of the beer auction houses as they attract beer consumers. In addition, the high level of biological pathologies at the mining site is maintained by inadequate treatments using drugs (mainly antibiotic therapy) consisting of self-medication (wrong medication and wrong dosage).

### Determinants of biomechanical health risks

Biomechanical health risks (with consequences as musculoskeletal disorders, fatigue, trauma, etc.) may result from the fact that diggers work with inappropriate equipment for long periods of time in uncomfortable postures in the mines. Furthermore, diggers are pushed to make extra hours of work searching for survival means due to poverty and the local perception of ‘virility’ according to which “men don’t get tired”. Enduring prolonged hours of work is a leading criterion for being selected as a digger irrespective of the associated biomechanical risks.

### Analysis of the importance given to pathologies, healthcare resources and constraints

Proportional piling is a tool that is effective in prioritizing local community participation activities [36]. It enabled the involvement of mining actors in examining the importance of health risks and pathologies, the constraints in the organization of healthcare in the mining site and collective initiatives that can be mobilized for the prevention of health risks.

### Frequencies and importance given to pathologies in the mining site

**Fig. 4:**
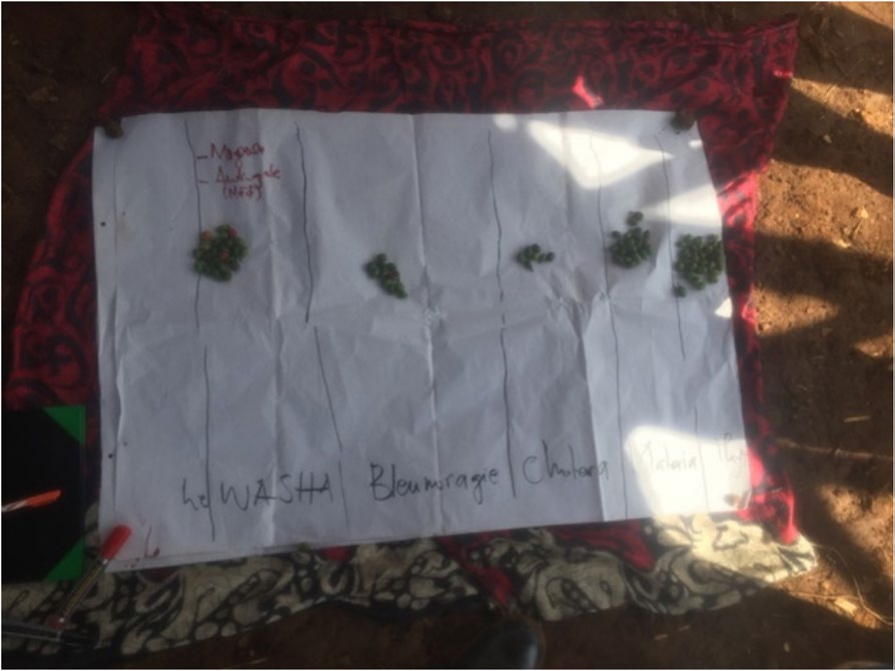
Picture of the proportional piling made on the most common diseases

The proportional piling illustrates that according to community perceptions, the most common diseases are STIs, including WASHA and gonorrhea (Fig. 4) followed by typhoid fever. Attention was given to health burden generated by the cholera epidemic experienced in the Luhihi mining camp in 2020.

### Constraints in the common illnesses treatment

**Fig. 5:**
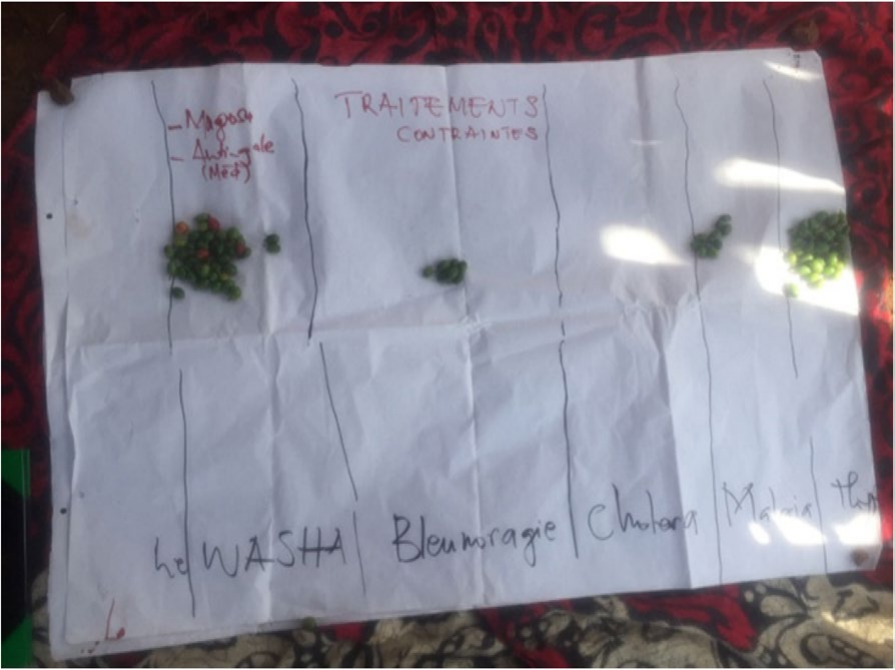
Picture of the proportional piling on the pathologies that are most likely to cause treatment difficulties

Some treatment constraints are associated with the unavailability and accessibility of drugs as well as disease recurrence. The pathologies that are mostly associated with treatment constraints are “washa” and typhoid fever (Fig. 5). It has been observed that medication were often associated with practices that do not comply to pharmacological indications. This has been the case for medication against gonorrhoea, which is however deemed as effective by patients. Diggers revealed that gonorrhea is treated at the site as following:

“...gonorrhea is nothing compared to WASHA, just a bottle of “Simba“ (strong alcoholic liquor) is enough to cure gonorrhea.” Extract from a digger/miner during a focus group. November, 2021 at Luhihi Mining Camp.

Also, a store owner of a drugstore told us the following:

“To treat gonorrhea, we give a mixture of half a bottle of the strong alcoholic liquor “SIMBA“, 10 capsules of chloramphenicol and one capsule of ampicillin. If it doesn’t work, we give the cefixime”. Extract from the semi-structured interview with a drug seller. November, 2021 at the Luhihi Mining Site.

**Fig. 6:**
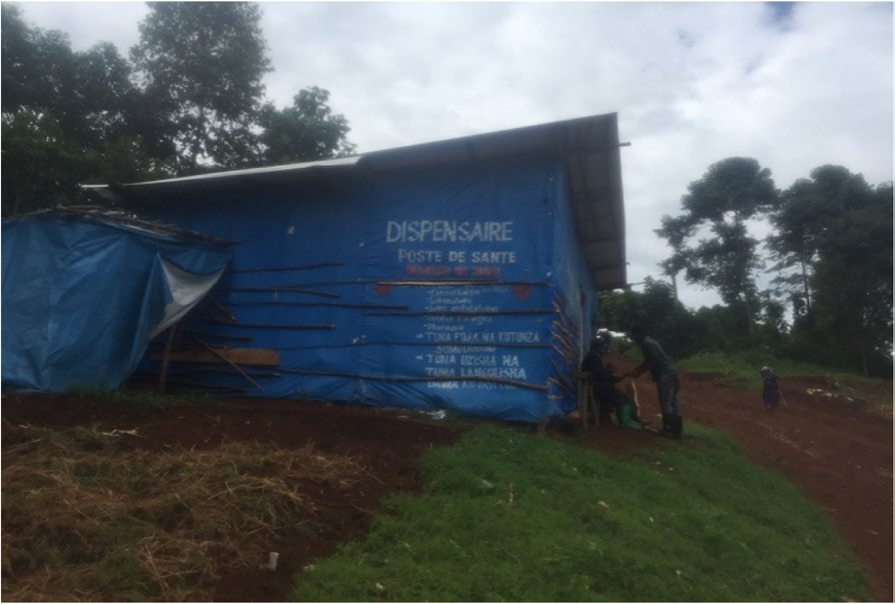
Picture of a dispensary of the Luhihi mining site

A dispensary of the Luhihi mining site named “Mungu ni jibu” meaning “God is the answer” (Fig. 6). It’s indicated that it provides medical consultancy, laboratory, ambulatory care, surgery, pharmacy and drugs wholesaling, and poison treatment.

### Sources of medical care resources at the mining site

**Fig. 7:**
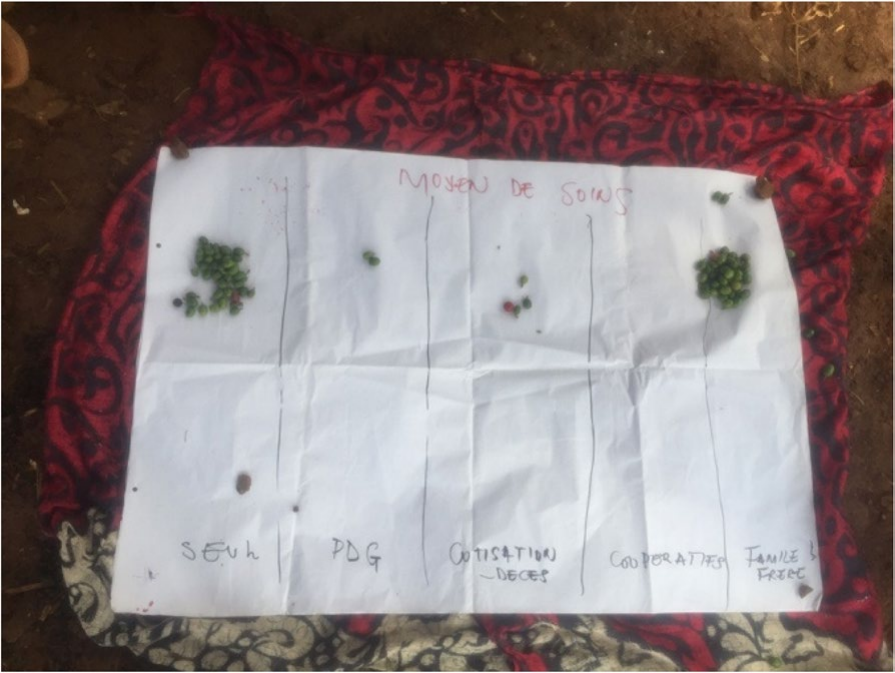
Picture of the proportional piling on the sources of medical care resources in the mining site

Mine diggers are the main actors in the gold mining activities (Table 5). Ideally, mine diggers use their individual resources and savings to pay for medical treatment. In case their individual resources and saving are limited, mine diggers require assistance from their respective families to pay for their medical fees (Fig. 7). In addition, the “ PDG “ and contributions between miners constitute alternative sources for health care.

### Opportunities of collective initiatives for the prevention of health risks

**Fig. 8:**
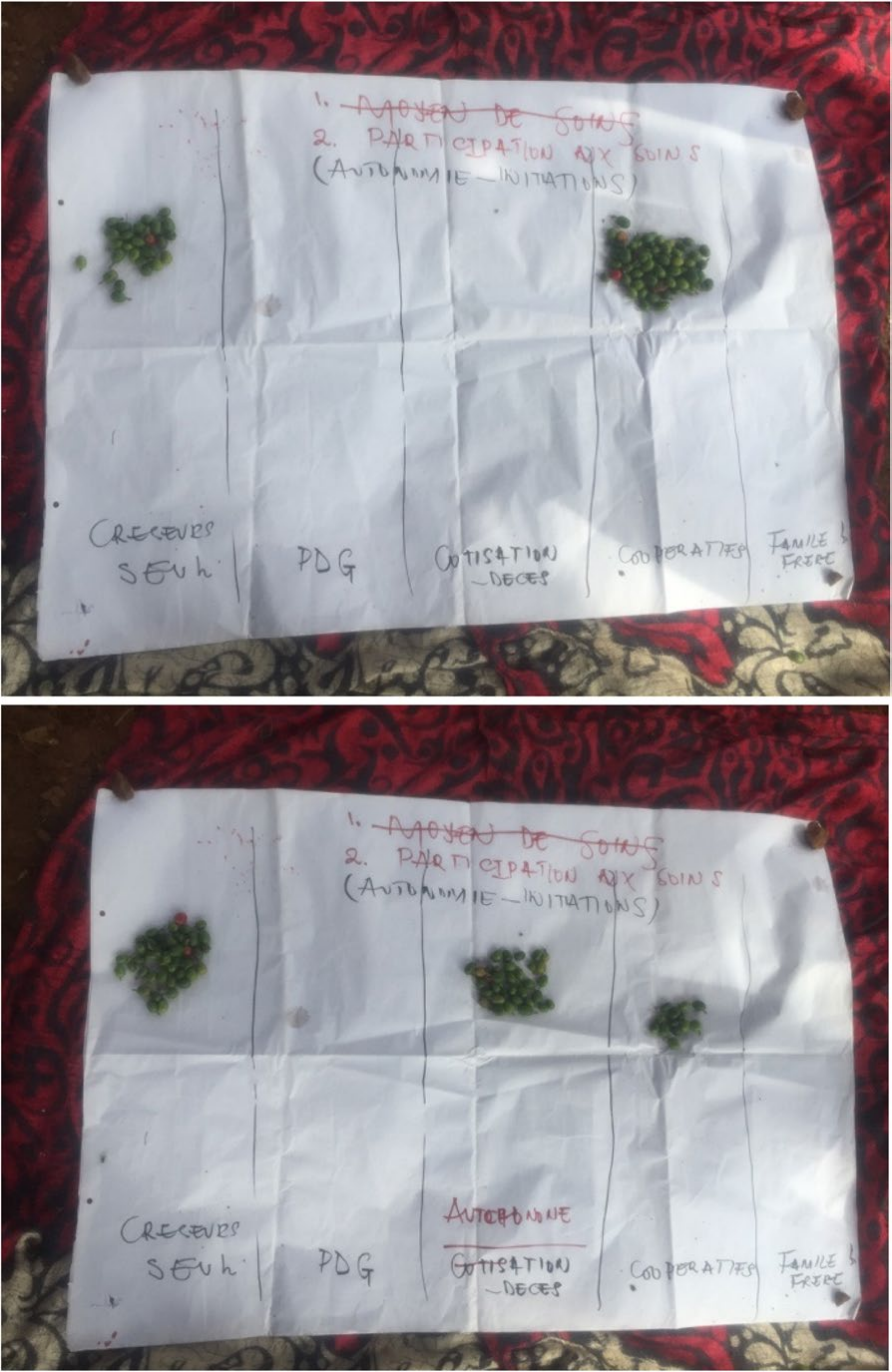
Pictures of the proportional piling made on opportunities of collective initiatives for the prevention of health risks

Mine actors have reported that diggers (Fig. 8) are in a better position to initiate revenue collection plans for sharing health care costs. Particular attention was given to the role of mining cooperatives (Fig. 8). These might encourage the “PDG” and miners to act altogether in collective structures enabling fundraising for health care. In addition, some informants revealed that indigenous people (Fig. 8) are in better positions to organize long-term collective actions given the fact that the other actors often leave the mining site after getting minerals. A Miner indicated his willingness to participate in cost-sharing initiatives for the prevention of health risks in this sense:

“As in the old mining carrier where I worked, we were able to make weekly contributions of 2,000 Congolese francs (equivalent to $1) or more depending on our agreement to help each other with health care...” Extract from a digger/miner during a focus group. November, 2021 at Luhihi Mining Camp.

In addition, a sex professional said the following:

“When a colleague is seriously ill, the miners contribute 1500 Congolese francs (equivalent to 0.75$) to pay for the transportation of the patient to join his family in the city for medical care”. Extract from a Sex professional during a focus group. November, 2021 at Luhihi Mining Camp.

## Discussion and perspectives

### Actors, health risks, and health care resources at the mining site

The actors in the Luhihi Mining site are on one hand the « PDG », the « Supporters », the Diggers called « demushimba », the Transportors, the person in charge of crushing and « washing », the economic dealers and sex professionals of called “Diggers without spade”. On the other hand, there are actors involved in the mining site administration such us mining agents, the camp Committee, the mining cooperative, the mine police. In the DR Congo, the artisanal mining sector faces considerable challenges including socio-economic problems, underutilization of production capacity, lack of state monitoring, and above all the activism of armed groups, some of whom control certain mining sites outright [37]. Formalization of the artisanal mining sector in the DRC is still at a basic stage. A few cooperatives are working, but are hampered by the fact that the informal sector offers more advantages to diggers than they can handle [15]. It was estimated that more than half of the cassiterite and coltan production and more than 90% of gold production in Eastern DR Congo is ‘informal’. This means that a large part of the exploitation and the export of mineral resources is not registered, nor regulated by the state and its legal framework [38]. This is due to factors including the importance of these activities for livelihoods and the lack of alternatives, the maladjustment of the law to local realities, and weak implementation of law due to the state’s limited capacities, and political will etc. [12, 38].

At the Luhihi artisanal mining site, biological health issues (STIs, malaria, diarrheal diseases, etc.) are perceived as having a high level of risk by the actors in the mining work place. In artisanal mining, the most common biological hazards affecting them are waterborne and vector-borne diseases, STIs, HIV/AIDs, and tuberculosis. Water and sanitation infrastructure are often limiting factors in artisanal and small-scale mining camps given that many sites are in remote locations that are hard to reach and the mining is often a transient activity [19].

Psychosocial and biomechanical hazards such as exposition to stress and fatigue are ranked second by mining players considering the levels of risk. In artisanal mining, biomechanical hazards such as heavy workloads, repetitive tasks, prolonged working hours and unsafe equipment can lead to the development of musculoskeletal disorders. Mostly recorded musculoskeletal disorders include shoulder disorders, fatigue and lower back pain among others [12]. Several studies have recorded drugs and alcohol among psychosocial hazard agents that affect both adult (mostly male) and child miners in artisanal mining. The migratory nature of many people who engage in ASGM is believed to contribute to drug and alcohol abuse which are seen as a way to cope with difficult circumstances [19].

The mine actors do not consider high risks from exposure to physical agents (such as heat, humidity, oxygen levels, loud noise, electricity, explosives, etc.) or chemical agents (such as mercury, silica, carbon monoxide, etc.), even though these agents are associated with mining practices. Some mine actors have stated that “they use mercury and coal in the processing of gold” (Extract from a PDG during interviews. November, 2021 at Luhihi Mining Camp). A study on artisanal mining in the DR Congo reported that miners barely access health care as the result of limiting factors incluging the instability of their incomes, the scarcity of health care facilities and shortage of qualified personnel in addition to the hardness of reaching most hospitals with are located far from most mines [15]. Using anecdotal evidence from the Kamituga gold mine, researchers have shown that the local population and miners are not aware of potential risks of using mercury because the associated adverse effects are not immediate. The overall consequence is that mercury remains a major but invisible threat to human health and the environment [18].

Constraints for treatment were related to the unavailability and inaccessibility of drugs in addition to disease recurrence. The main mining actors consider their individual resources as their primary means of treatment, followed by assistances from their respective families. In addition, they have confirmed their willingness for the prevention of health risks through participation in cost-sharing initiatives. These constitute an enormous progress for the development of sustainable risk prevention services based on the resources of mining actors. These individual, contextual and relational resources mobilized by mining actors constitute assets for the development of services. Examining the resurgence role, Village Development Associations in Cameroon, Fonchingong and Fonjong found that community members are increasingly shouldering the adverse consequences of the economic downturn and the growing inability of the state to provide economic and social development by initiating, mobilizing and galvanizing their own resources with the aim of improving their standards of living [39].

### Grid of powers and interests in relation to mobilization of resources for prevention and treatment of health risks

We used the “power/interest grid” (Table 7) to translate the results of the study by classifying all the actors that are affected by the health risks in the artisanal mining and those who are interested and capable of mobilizing resources in the perceptive of actions for change [40].

**Table 7:** Power and interest Grid

Ttype of actors	Specifications
**Actors with Low power/low interest: these actors are currently of minor importance for resource mobilization**	**Health zones, natives farming populations, non-governmental organizations. (i) State health zones do not have financing plans that include mine actors, (ii) faming natives consider the mine as a socio-financial threat, (iii) mine actors consider that NGOs act on an ad hoc basis rather than on a long-term basis.**
**Actors with “Low power/high interest”: although these actors have little power, they can be important stakeholders in resource mobilization.**	Minors, Sex professionals and Economic actors are under-resourced but have a high interest in health care. They are constantly financing themselves their health care. They are also willing to participate in cost-sharing initiatives for the prevention of health risks by releasing the amounts agreed upon or another reasonable amount.
**Actors with “High power/low interest”: this group is the most challenging to address. Strategies are needed to be settled in order to engage them.**	Mining Cooperatives, State agencies and Mine Police. (i) Firstly, mining cooperatives have the power to initiate but do not promote the health actions of their members. (ii) Secondly, State agencies do not encourage cooperatives, PDGs to provide their members with health protection tools, the establishment of health care structures as well as the creation of health mutuality. (iii) The mine police are capable of enforcing the application of preventive measures of health risks but do not do so.
**Actors with “High power/high interest”: these are the actors with whom one might clearly engage in resource mobilization**	“PDG” and “supporters” have the potential to assist miners in paying for their health care. They also have an interest in enhancing miners’ health as they are exploiting for the account of the “PGDs and Supporters”.

Artisanal miners living in the Eastern DR Congo are encouraged by the government to gather into mining cooperatives, in line with formalization policies [8]. According to the legal framework for mining cooperatives in the DR Congo, a mining cooperative is a group of artisanal miners, approved by the Minister, and engaged in artisanal mining of minerals or quarry products within a certified artisanal mining zone [28]. The Mining Code of the DR Congo refers the training and technical supervision of operators as well as the implementation of these safety and hygiene standards in the artisanal mining to state services such as SAEMAPE (Artisanal and Small-Scale Mining Assistance and Support Service) and the mining cooperatives.

### Biological and biomechanical health risks

The challenge of implementing management options for biological pathologies (cholera, malaria, thyphoid fever, etc.) and health outcomes of biomechanical hazards (shoulder disorders, lower back pain, chronic injuries, fractures, eye injuries, etc.) is primarily organizational. To prevent these risks, the various actors of the mine have different levels of responsibilities. However, the miner code and the pontential ressourses attribute more acting power to state agencies such as the SAEMAPE, the mining cooperatives and the owners of the mining shafts (“PDG” and “Suporteurs”). These are able to set up sanitary facilities, to take care of the hygiene and sanitation, to guarantee the supply of potable water through alternative sources as reservoirs, wells, boreholes, river water treatment, etc. In addition, these actors can guarantee access to basic health care services in the mines by providing adequate equipment to mobile clinics. It is mandatory to provide for mechanized drilling and heavy transport equipment and protection against rockfall and explosions. The above mentioned actors, have the pontential to oraganize prevention services of biological and biomechanical health risks through mobilization of financial means and regulation of services in mining sites. The others actors of the mining site (diggers, sex professionals, shopkepers, etc.) can adopt these health care initiatives given that they have declared in this study, that they are highly interested in preventing these risks. They can also have access to sanitary services as they are constatly financing their sanitary care themselves.

### Chemical and physical health risks

In addition to the organizational challenges outlined above and the responsibilities of actors, there is a need of awakening awareness among artisanal miners about the harmful effects of the chemicals and physical hazards to which they are exposed. This study highlights the exposure of miners to mercury, carbon monoxide, heat, humidity, low levels of oxygen, loud noise, electricity, etc. Evidence from studies on long-term health effects of these hazards should support the organization of outreach and engagement activities with mining actors. Furthermore, personal experiences of mine workers on health risk effects such as chronic pulmonary diseases, tuberculosis, insomnia, gastrointestinal disorders, headaches, etc. should be used to raise awareness and promote the adoption of the most accessible protective measures. These include wearing masks, reducing humidity and ensuring the level of oxygen in the underground pipes, etc. Nevertheless, the application of these measures would be threatened by the weariness of the actors to support its financial cost in the medium and long term. Thus, to ensure that they are applied continuously, the state agencies in the mining will have to set incentives.

### Psychological health risks

In order to intervene on the health effects of psychological hazards (such as the stress leading to drug and alcohol abuse, fatigue, etc.), actions to respond to socio-economic constraints of the mine workers must be taken in addition to the organizational and awareness-raising measures mentioned above. Due to the lifestyle factors of the workers (such as the transient, poverty, separation from family, long working hours, heavy workloads, loss of work due to injury, etc.) they need to benefit from pyschological follow-up based on their socio-anthropological context. They should also be trained on how to save money in order to make efficient use of it. These activities should be integrated into the training tasks that the mining cooperatives are required to perform by the legal framwork for ASM activities in DR Congo.

### Dialogue for change regarding mine actors’ ignorance of exposure to physical and chemical health risks

Although we recorded the use of mercury in gold panning at the Luhihi mine, the miners seemed not to be aware of the health risks that it poses. After the Minamata Convention (MC) on mercury (Hg) control came into force, the demand for Hg in the ASGM sector may not have decreased, thus driving the increase in the Hg trade through illegal routes such as mislabeling, smuggling, or black markets [41].

Participatory behavioral change research, awareness, and regulatory incentives are needed as key actions for addressing artisanal mine actors’ ignorance of the danger of exposure to chemical hazards such as mercury. Firstly, these actions will aim at delivering appropriate messages to drive change. This requires identifying target audiences, formulating key messages, adapting messages to the roles that different stakeholders may play. It also entails informing through accessible communication channels, incorporating ethical considerations, and integrating interactive communication by considering feedback from target audiences. Secondly, to ensure the message of the communications remain in the memories of the target audiences and raise their conscience, the awareness activities should be based on the “dialogue for change” pedagogy [26]. This proposes a communication structure with several activities including contacting and catching attention of the targets, sharing experiences of side effects, the activities for explicit awareness, and the communication maintenance activities. Thirdly, to ensure the motivation of mining actors, these change projects must integrate biological risks (infectious diseases) that mining actors perceive to be at a higher level of risk than chemical health risks.

### Are artisanal mining stakeholders capable of organizing health care services?

In the artisanal mining site, where monetary flows are certain, stakeholders must take in charge the health care cost in order to prevent health risks such as the cholera epidemic that occurred in the Luhihi mining site in 2020. A model for the provision of health care services and responsibilities have to be established between mine actors. Within the model the state agencies should set on regulations for the control of the implementation of health care services. From the observations on the Luhihi mine case study, it is clear that there is a need to strengthen the provision of health care factors, including materials, infrastructure, personnel, services, drugs, water supply, hygiene and sanitation. There is a need of organizing a financing scheme, which can be public financing or actors gathered in mutual or private insurance companies. The health care demand and the financing scheme have to integrate socio-economic, financial and cultural accessibility determinants.

## Conclusion

This article argues that the adoption of positive practices involves many socio-economic and administrative aspects that may limit artisanal miners’ adherence and access to the means of health risk prevention practices. This transdisciplinary essay brought together scientists and people concerned with the health risk issue in the Luhihi gold mine, in South Kivu. It recorded that biological risks and psychosocial risks are perceived as having a high level of risk by the actors in the mining work place. The most constraints for treatment of the common illnesses were associated with the unavailability and inaccessibility of health services, drugs as well as the disease recurrence. As to prevent these, the actors of the mine system have to set up sanitary facilities, to take care of the hygiene and sanitation as well to ensure the use of mechanized drilling and heavy transport and protection equipments. Although, mine actors do not perceive a high risk from exposure to physical agents (heat, humidity, oxygen levels, etc.) and chemical agents (exposure to mercury, silica, carbon monoxide), these are closely related to their mining practices. For addressing these ignorance, participatory behavioral change research, awareness, and regulatory incentives are needed as key actions. The main source for medical treatment is the individual resources of mine diggers. However, we have recorded that more acting power for organising health services is attributed to the state agencies, the mining cooperatives and the owners of the mining shafts.

The originality of this paper is to complete the exiting literature on health risks in artisanal mining by translating its finding into perceptives. These included the grid of powers and interests in relation to the mobilization of resources for the prevention and treatment of health risks. The contribution to the dialogue for change regarding the ignorance of exposure to chemical risks by the actors of the mine was also taken into consideration. In addition, an analysis of the ability of artisanal mining actors in the DR Congo to organize health risk prevention services in the mining sector was performed.

## Data Availability

The datasets used and/or analyzed during the current study are available from the corresponding author on reasonable request.

## Abbreviations

ASM: Artisanal Small-Scale Mining
NGOs: Non gouvernemental Organisations
HIV/AIDS: Human immunodeficiency Virus / Acquired immunodeficiency syndrome
STIs: Sexually Transmitted infections
FC: Focus Group
